# Influence of Hospital Ownership on Patient Outcomes in ST-Elevation Myocardial Infarction-Induced Cardiogenic Shock Undergoing Revascularization: A Retrospective Cohort Study

**DOI:** 10.7759/cureus.84471

**Published:** 2025-05-20

**Authors:** Mushrin Malik, Juan C Sequeira Gross, Garry Francis-Morel

**Affiliations:** 1 Internal Medicine, St. Barnabas Hospital Health System, New York, USA; 2 Internal Medicine, Family Health Center of Southwest Florida, Fort Myers, USA; 3 Pulmonary and Critical Care, Saint Louis University School of Medicine, Saint Louis, USA

**Keywords:** acute coronary syndrome, health public, left main occlusion with cardiogenic shock, primary pci, private hospitals

## Abstract

Background

The healthcare landscape is notably divided between investor-owned and nonprofit hospitals, raising questions about the impact of hospital ownership on patient outcomes, especially for high-stakes conditions such as cardiogenic shock resulting from ST-elevation myocardial infarction (STEMI) treated with percutaneous coronary intervention (PCI). This study analyzed whether differences in hospital ownership are associated with variations in mortality, length of stay (LOS), and healthcare costs.

Methodology

We conducted a retrospective cohort study using the National Inpatient Sample (NIS) from 2016 to 2021, identifying 95,260 adult patients with STEMI-induced cardiogenic shock treated with PCI. Identification was based on validated International Classification of Diseases, Tenth Revision, Clinical Modification coding algorithms. The primary outcome was in-hospital mortality, with LOS and total hospital charges (inflation-adjusted via the Consumer Price Index) as secondary outcomes. All outcomes were assessed using multivariable regression models adjusting for patient demographics (age, sex, race/ethnicity), comorbidities, insurance type, and hospital-level factors (region, teaching status, and bed size). Regional cost variation and case mix were also accounted for. Propensity score matching was additionally performed to validate results.

Results

The analysis revealed no significant difference in mortality rates between investor-owned (27.22%) and nonprofit hospitals (26.93%, adjusted odds ratio (aOR) = 1.03, p = 0.60; 95% confidence interval (CI) = 0.91-1.16). Propensity score-matched analysis confirmed similar findings (aOR = 0.98, p = 0.696). Investor-owned hospitals, however, incurred significantly higher healthcare costs (average charges = $325,543 vs. $222,528; p < 0.001). The average cost difference of $103,015.50 remained statistically and systemically significant after adjustment and may reflect differences in resource utilization and/or billing practices. LOS was slightly shorter in investor-owned hospitals (6.51 vs. 7.27 days); while statistically significant (p < 0.001), this difference was not clinically meaningful after adjustment (coefficient = 0.19, p = 0.334). Key demographic and clinical predictors included age, insurance status, comorbidity index, hospital bed size, and teaching status. Racial and insurance-based disparities, particularly among Hispanic patients and Medicaid enrollees, were associated with higher costs, though not fully explained by hospital ownership.

Conclusions

In this national analysis, hospital ownership was not associated with differences in mortality for STEMI-induced cardiogenic shock treated with PCI, but was independently associated with substantially higher hospital charges in investor-owned hospitals. These findings demonstrate association, not causation, and highlight the need for future research into cost drivers and initiatives to promote high-value, standardized care across all hospital types.

## Introduction

Cardiogenic shock (CS), characterized by cardiac impairment leading to systemic hypoperfusion and decreased tissue oxygenation, stands at the forefront of clinical emergencies owing to its association with acute myocardial ischemia and infarction (AMI). This critical condition is the leading cause of in-hospital mortality among AMI patients, highlighting a pivotal area of concern in cardiovascular medicine [1]. Annually, CS affects 5% to 10% of all AMI patients in the United States [2-5], with a staggering 30-day mortality rate of nearly 40%, which escalates to approximately 50% within a year [5-8].

The American Hospital Association reports that, as of 2024, there are 6,120 hospitals in the United States, comprising 2,987 nonprofit and 1,219 for-profit institutions [9]. These data underscore the significant presence of for-profit and investor-owned hospitals, including those acquired by private equity, in the U.S. healthcare system. Research by Bruch et al. reveals that hospitals acquired by private equity experience notable gains, including an increase in net annual income of approximately $2.3 million and an additional $407 in the total charge per patient per day [10]. Although these trends were reported in the early 2020s, they reflect financial strategies that may have influenced billing patterns during the 2016-2021 study period. Bain & Co.’s 2022 report further supports the sustained momentum of private equity activity in healthcare, with a disclosed deal value of $90 billion, despite a decline from previous years [11]. Moreover, private equity-backed hospitals are known to reduce or eliminate less profitable services, such as organ transplants or trauma care, and may prioritize billing optimization over resource expansion [12,13].

This study aimed to address a gap in the literature by evaluating how hospital ownership models, specifically nonprofit versus for-profit status, correlate with patient outcomes and hospital charges in cases of CS secondary to ST-elevation myocardial infarction (STEMI). While prior work has identified financial behavior changes in for-profit systems, how such financial orientation affects outcomes in high-acuity cardiovascular emergencies, such as STEMI-induced CS, remains underexplored. Given the clinical urgency and complexity of CS management, and the resource-intensive nature of interventions such as percutaneous coronary intervention (PCI), concerns have emerged about whether profit-driven care models compromise or inflate resource use without necessarily improving outcomes.

PCI, the recommended standard of care for STEMI [14], is a high-cost, high-value procedure with average hospitalization charges ranging from $20,000 to over $90,000 depending on clinical complexity, regional pricing, and payer mix [15]. Therefore, understanding how hospital ownership influences total charges and patient outcomes in this population may provide insights into systemic inefficiencies and opportunities for cost standardization.

This study focuses on the broader for-profit hospital category (which includes but is not limited to private equity ownership) and investigates the association, not causation, between ownership status and three key outcomes, namely, in-hospital mortality, length of stay (LOS), and total hospital charges. We hypothesized that investor-owned hospitals may exhibit higher adjusted costs without demonstrable differences in clinical outcomes due to variations in administrative, financial, and care delivery practices. Utilizing data from six years of the National Inpatient Sample (NIS), our analysis focuses on potential disparities in patient care and hospital charges across ownership types, with the aim of generating hypotheses for future research and informing value-based care policy discussions.

## Materials and methods

Data source

This retrospective cohort analysis adhered to the Strengthening the Reporting of Observational Studies in Epidemiology (STROBE) guidelines and utilized the NIS from the Healthcare Cost and Utilization Project (HCUP), a collaboration between the Federal Government, state governments, and the private sector, sponsored by the Agency for Healthcare Research and Quality (AHRQ). As the largest publicly available inpatient database in the United States, the NIS contains data from 1988 onward, covering 48 states and the District of Columbia [14]. The analysis excluded long-term acute care hospitals and rehabilitation centers, focusing on over seven million annual hospital stays. All data are de-identified and publicly available, allowing for national estimates using discharge weights. All researchers adhered to HCUP data use agreements and regulatory standards.

Study population

We identified patients aged ≥18 years hospitalized between January 2016 and December 2021 with a primary diagnosis of CS secondary to STEMI and treated with PCI using either drug-eluting stents, bare-metal stents, or balloon angioplasty without stent placement. Inclusion was based on International Classification of Diseases, Tenth Revision, Clinical Modification (ICD-10-CM) codes. A supplemental table listing all ICD-10 codes used in the study has been included in the Appendices.

Missing data

Key variables such as race, mortality, and insurance status had missing data, with rates comparable to prior NIS studies, except for race in 2016. We applied listwise deletion, excluding patients with incomplete records from each outcome model. This method is consistent with prior NIS analyses and was chosen for its transparency and ease of implementation; however, we acknowledge that listwise deletion assumes data are missing completely at random (MCAR). No sensitivity analyses were conducted to test this assumption, which may introduce selection bias and is a limitation of our approach. The total number and percentage of excluded records were recorded and are reported in the Results section.

Study outcomes

The primary outcome was in-hospital mortality. Secondary outcomes included LOS, defined as the number of days from admission to discharge or death, and total hospital charges, expressed in U.S. dollars. Charges were adjusted for inflation using the Consumer Price Index (CPI). Cost data reflect hospital charges rather than actual resource utilization; no cost-to-charge ratios were applied. To account for regional variation and hospital-level pricing differences, geographic and structural covariates were included in the adjusted models.

Statistical analysis

Statistical analysis was conducted using STATA version 18.0 (StataCorp, College Station, TX, USA), incorporating the complex sampling design of the NIS, i.e., stratification, clustering, and weighting, to produce nationally representative estimates. Descriptive statistics were calculated using chi-square tests for categorical variables and t-tests for continuous variables.

We used propensity score matching (PSM) to reduce confounding when comparing patients treated at investor-owned versus nonprofit hospitals. A non-parsimonious logistic regression model was used to estimate the propensity scores based on patient-level covariates, including age, sex, race/ethnicity, insurance type, and comorbidities (e.g., diabetes with complications, congestive heart failure, chronic obstructive pulmonary disease (COPD), cerebrovascular disease, hemiplegia, hypertension, connective tissue disease, hyperlipidemia, and obesity), as well as hospital-level characteristics (bed size, region, and teaching status). Matching was performed using a 1:1 nearest-neighbor method without replacement. Covariate balance was assessed using standardized mean differences, as shown in the supplementary figure provided in the Appendices.

For outcome comparisons, we employed multivariable regression models. Logistic regression was used for binary outcomes (mortality), and generalized linear models were applied for continuous outcomes (charges and LOS). For cost analyses, a gamma distribution with a log link was specified due to right-skewed data; no log transformation was applied to raw charges. Model covariates included patient demographics, hospital characteristics (bed size, teaching status, region), insurance type, and the Charlson Comorbidity Index (CCI). All models were adjusted for inflation and regional cost variation.

To minimize false discovery from multiple testing, we applied the Bonferroni correction, adjusting for the number of pairwise comparisons in each outcome domain. While conservative, Bonferroni was chosen to ensure rigorous control of Type I error in this exploratory, hypothesis-generating study [16].

Patient confidentiality and institutional board review

This study used HCUP-limited datasets with 16 direct identifiers removed in accordance with the HIPAA Privacy Rule, rendering it exempt from institutional review board review. The analysis adhered to ethical standards outlined by the World Medical Association Declaration of Helsinki.

## Results

Patient’s characteristics

**Figure 1:**
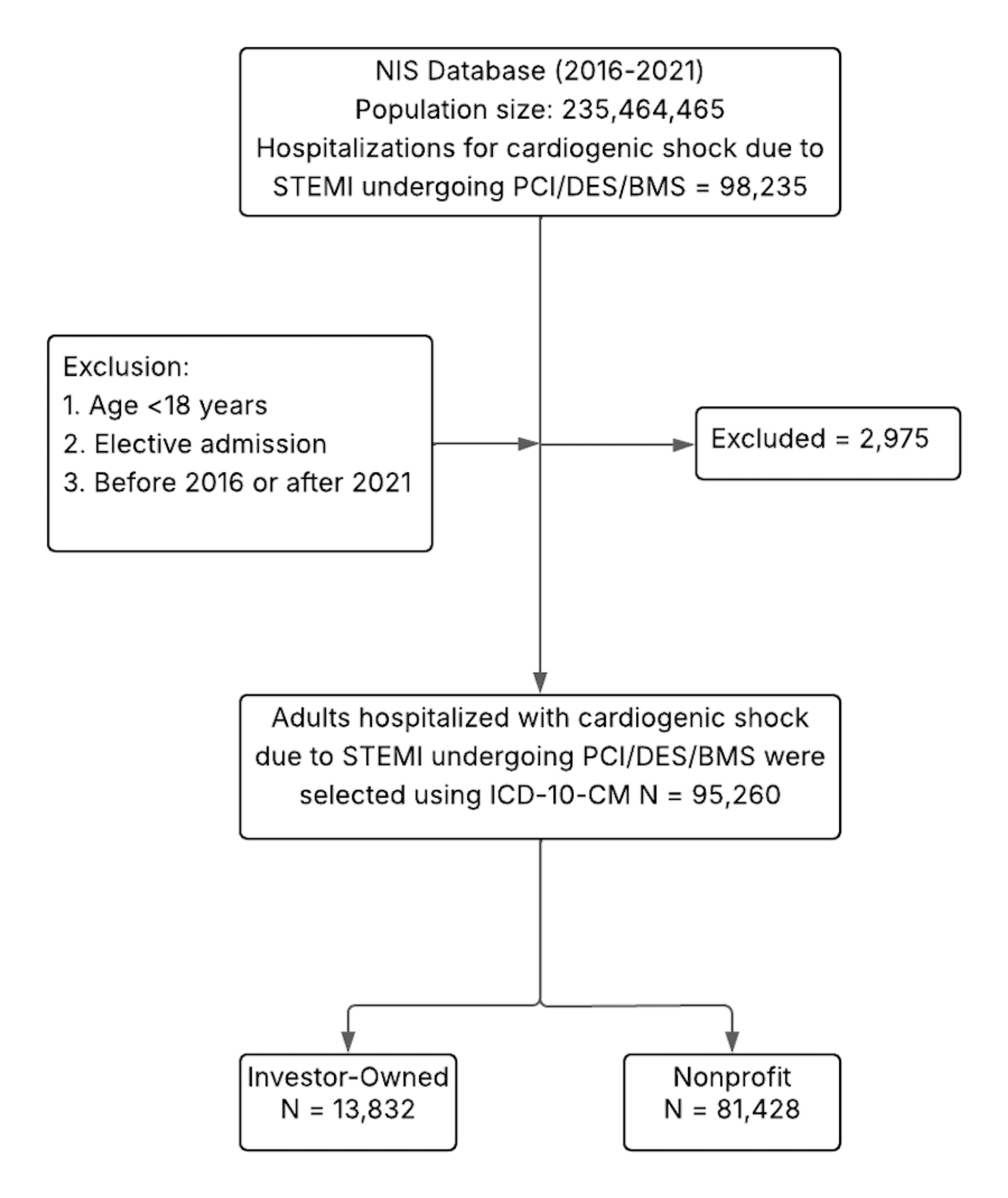
Study flowchart. NIS = National Inpatient Sample; STEMI = ST-elevation myocardial infarction; PCI = percutaneous coronary intervention; DES = drug-eluting stent; BMS = bare-metal stent

The flowchart (Figure 1) illustrates the patient selection process from the NIS database for the years 2016-2021. Initially, 235,464,465 hospitalizations were considered. We identified 98,235 hospitalizations for CS due to STEMI treated with PCI using drug-eluting stents or bare-metal stents. The exclusion criteria, i.e., patients under 18 years of age, elective admissions, and admissions outside the study timeframe, led to the removal of 2,975 records, resulting in a final analytic sample of 95,260 adult hospitalizations with ICD-10-CM-coded STEMI-induced CS treated with PCI. Transfers between hospitals could not be reliably tracked in NIS and were therefore not separately excluded; this is acknowledged as a limitation. The final sample was stratified by hospital ownership: 13,832 (14.52%) patients were treated in investor-owned hospitals and 81,428 (85.48%) in nonprofit institutions.

Table 1 presents baseline characteristics stratified by hospital ownership. While these are unadjusted comparisons, all variables included were later adjusted for in multivariable models. The mean age was slightly higher in investor-owned hospitals (66.17 vs. 65.68 years, p = 0.082), and sex distribution was similar (67.92% male in investor-owned vs. 67.43% in nonprofit, p = 0.648).

**Table 1:** Demographic characteristics of patients admitted for cardiogenic shock due to ST-elevation myocardial infarction. Comparisons were made using chi-square tests for categorical variables and independent t-tests for continuous variables. Chi-square values ranged from 3.57 × 10⁵ to 345.41; t-values ranged from 2.55 to 16.89. A p-value <0.05 was considered statistically significant. PI = Pacific Islander; N/A = not applicable; N = number of patients; LOS = mean length of stay in days; total hospital charges = mean total charges incurred during hospitalization (USD)

Parameters	Total	Investor-owned (n = 13,832)	Nonprofit (n = 81,428)	P-value
Number of cases	95,260	14.52	85.48	N/A
Mean age (years)	65.75	66.17	65.68	0.082
Sex (%)				
Male	67.5	67.92	67.43	0.648
Female	32.5	32.08	32.57	
Race (%)				
White	73.8	70	74.45	<0.001
Black	7.68	5.96	7.97	
Hispanic	9.42	14.36	8.57	
Asian/PI	4.24	3.51	4.36	
Native American	0.71	0.72	0.71	
Others	4.15	5.47	3.93	
Insurance (%)				
Medicare	52.87	54.39	52.62	0.004
Medicaid	11.33	9.85	11.58	
Private	29.52	28.13	29.75	
No insurance	6.27	7.63	6.05	
Median household income (%)				
$1–$49,999	27.37	32.28	26.54	<0.001
$50,000–$64,999	27.42	27.55	27.39	
$65,000–$85,999	24.31	23.42	24.45	
≥$86,000	20.9	16.75	21.61	
Charlson Comorbidity Index (%)				
1	16.63	17.97	16.41	<0.001
2	31.34	29.68	31.62	
3	52.03	52.35	51.98	
LOS	7.16	6.51	7.27	<0.001
Hospital charges	231,867	315,640	217,749	<0.001
Died during hospitalizations (%)	26.97	27.22	26.93	0.772

Racial and ethnic distributions differed significantly (p < 0.001). White patients made up 73.8% overall, but a higher proportion were treated in nonprofit hospitals (74.45%) than in investor-owned ones (70%). Hispanic patients were more commonly treated in investor-owned hospitals (14.36% vs. 8.57%). “Others” includes multiracial, unknown, and unclassified race categories in the NIS dataset.

Insurance status varied (p = 0.004). While Medicare was most common across both groups, Medicaid patients were more frequently seen in nonprofit hospitals. Socioeconomic status, approximated by household income, also differed significantly (p < 0.001), with more low-income patients (<$50,000) in investor-owned hospitals.

The CCI distribution showed that patients with higher comorbidity burden (CCI ≥ 2) were more likely to be treated in nonprofit hospitals (p < 0.001), which could influence outcomes and was adjusted for in regression analyses.

**Figure 2:**
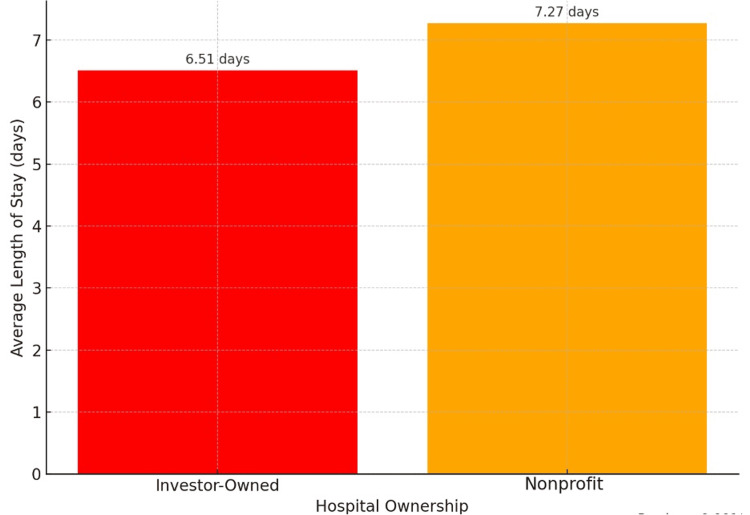
Length of stay by hospital ownership.

**Figure 3:**
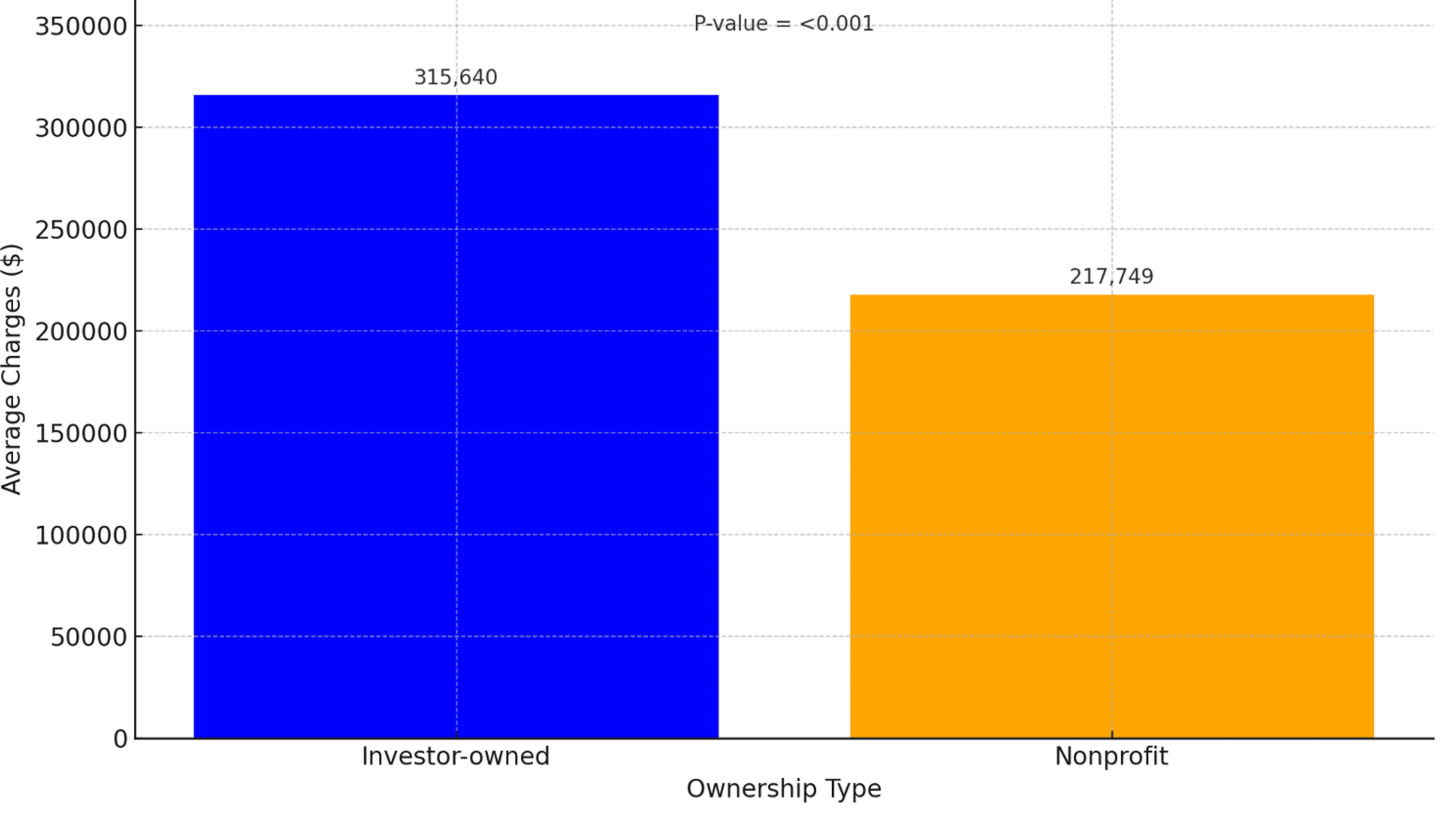
Hospital charges by hospital ownership.

Figure 2 shows a shorter mean LOS in investor-owned hospitals (6.51 days) compared to nonprofit ones (7.27 days) (p < 0.001). Although statistically significant, this 0.76-day difference may be of limited clinical relevance. Figure 3 displays hospital charges, which were markedly higher in investor-owned hospitals (mean = $315,640) than in nonprofit facilities (mean = $217,749) (p < 0.001). These are inflation-adjusted billed charges, not actual costs or resource use.

**Figure 4:**
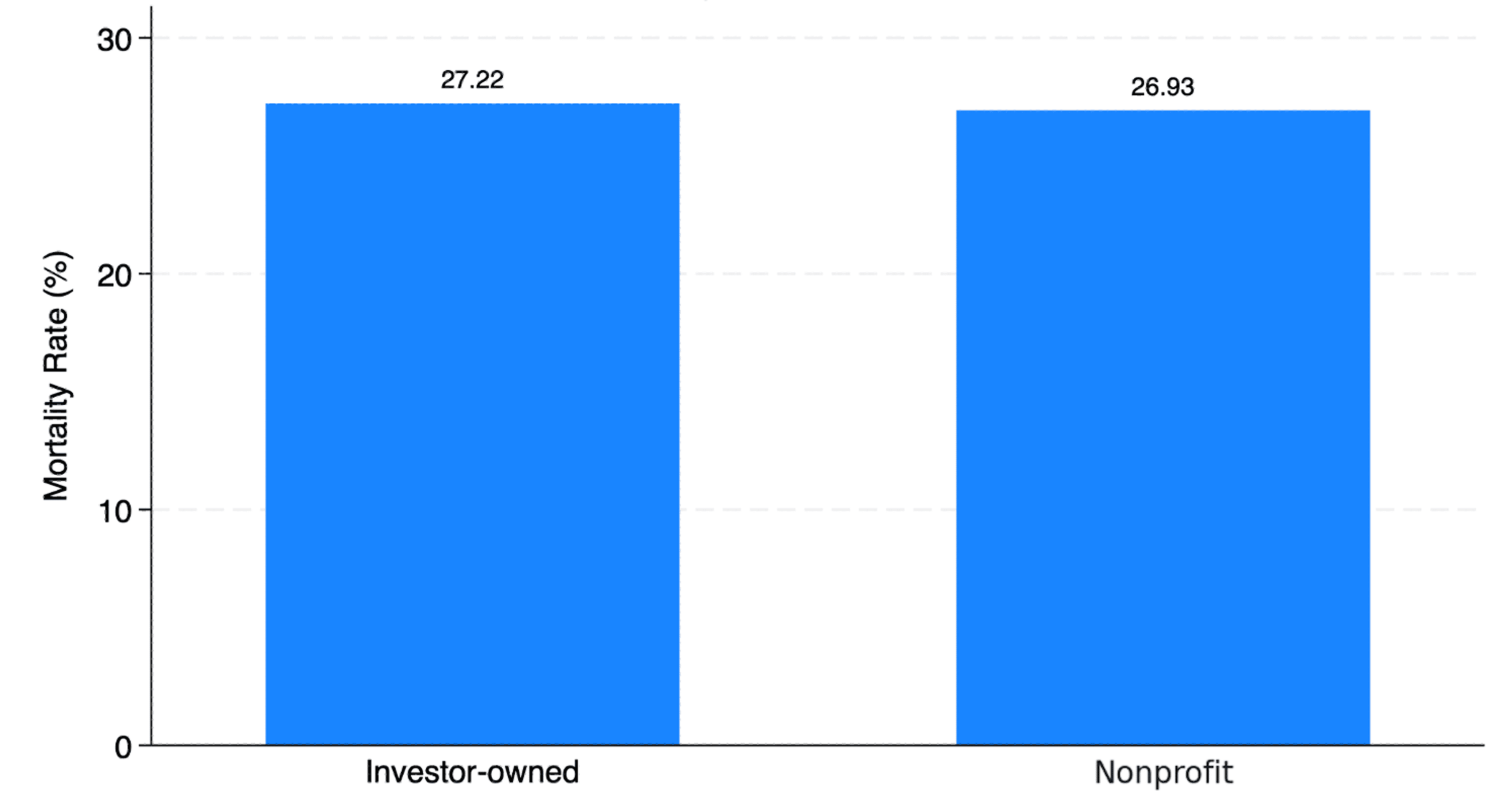
Mortality rate by hospital ownership.

Mortality rates were similar across ownership types (Figure 4), with 27.22% in investor-owned vs. 26.93% in nonprofit hospitals (p = 0.772). These unadjusted rates were further analyzed in multivariate models.

Patient’s comorbidities

**Figure 5:**
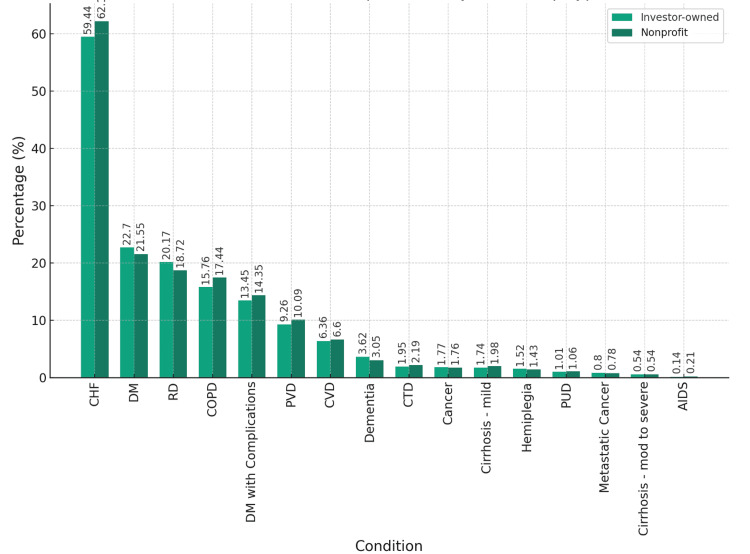
Comorbidities and complications differentiated by hospital ownership. The bar chart illustrates the percentage of patients with various comorbidities and complications differentiated by hospital ownership type: investor-owned versus nonprofit. CHF = congestive heart failure; PVD = peripheral vascular disease; CVD = cerebrovascular disease; COPD = chronic obstructive pulmonary disease; CTD = connective tissue disease; RD = renal disease; PUD = peptic ulcer disease; DM = diabetes mellitus; AIDS = acquired immunodeficiency syndrome

Comorbidities (Table 2, Figure 5) were generally similar between groups. Congestive heart failure was slightly less prevalent in investor-owned hospitals (59.44% vs. 62.17%) (p = 0.021). COPD approached significance (p = 0.052); however, after Bonferroni correction, it was not considered statistically significant. Nonetheless, these conditions may have clinical relevance given their impact on outcomes. For example, renal disease was 1.45% more common in investor-owned hospitals (20.17% vs. 18.72%) (p = 0.129), a potentially meaningful difference given the prognostic significance of renal disease. Sample size imbalance (13,832 vs. 81,428) may affect statistical power for subgroup analyses. Therefore, caution is warranted in interpreting borderline differences.

**Table 2:** Comorbidity profile of patients admitted for cardiogenic shock due to ST-elevation myocardial infarction. Comparisons were made using chi-square tests for categorical variables. A p-value <0.05 was considered statistically significant. CHF = congestive heart failure; PVD = peripheral vascular disease; CVD = cerebrovascular disease; COPD = chronic obstructive pulmonary disease; CTD = connective tissue disease; RD = renal disease; PUD = peptic ulcer disease; DM = diabetes mellitus; AIDS = acquired immunodeficiency syndrome

Comorbidities (%)	Investor-owned (n = 13,832)	Nonprofit (n = 81,428)	P-value
CHF	59.44	62.17	0.021
PVD	9.26	10.09	0.217
CVD	6.36	6.6	0.685
Dementia	3.62	3.05	0.167
COPD	15.76	17.44	0.052
CTD	1.95	2.19	0.491
RD	20.17	18.72	0.129
PUD	1.01	1.06	0.856
Cirrhosis - mild	1.74	1.98	0.441
Cirrhosis - moderate to severe	0.54	0.54	0.990
DM	22.7	21.55	0.250
DM with complications	13.45	14.35	0.284
Hemiplegia	1.52	1.43	0.776
Cancer	1.77	1.76	0.977
Metastatic cancer	0.8	0.78	0.942
AIDS	0.14	0.21	0.566

Hospital characteristics

Table 3 summarizes hospital-level characteristics. More patients in investor-owned hospitals were treated in small (28.27%) and medium-sized (39.8%) facilities compared to nonprofit hospitals, which had a greater share of large hospitals (60.87%) (p < 0.001). These are patient-weighted characteristics (i.e., based on where patients were treated).

**Table 3:** Hospital characteristics associated with admissions for cardiogenic shock due to ST-elevation myocardial infarction. Comparisons were made using chi-square tests for categorical variables. Statistical significance between investor-owned and nonprofit hospitals was determined using p-values, with a value less than 0.05 indicating a statistically significant difference.

Parameters	Total	Investor-owned (n = 13,832)	Nonprofit (n = 81,428)	P-value
Relative bed size (%)				
Small	14.38	28.27	12.02	<0.001
Medium	28.95	39.8	27.11	
Large	56.67	31.92	60.87	
Teaching status (%)				
Non-teaching	24.92	41.76	22.06	<0.001
Teaching	75.08	58.24	77.94	
Hospital region (%)				
Northeast	15.81	3.62	17.88	<0.001
Midwest	23.38	9.62	25.72	
South	37.99	59.11	34.4	
West	22.82	27.66	22	

**Figure 6:**
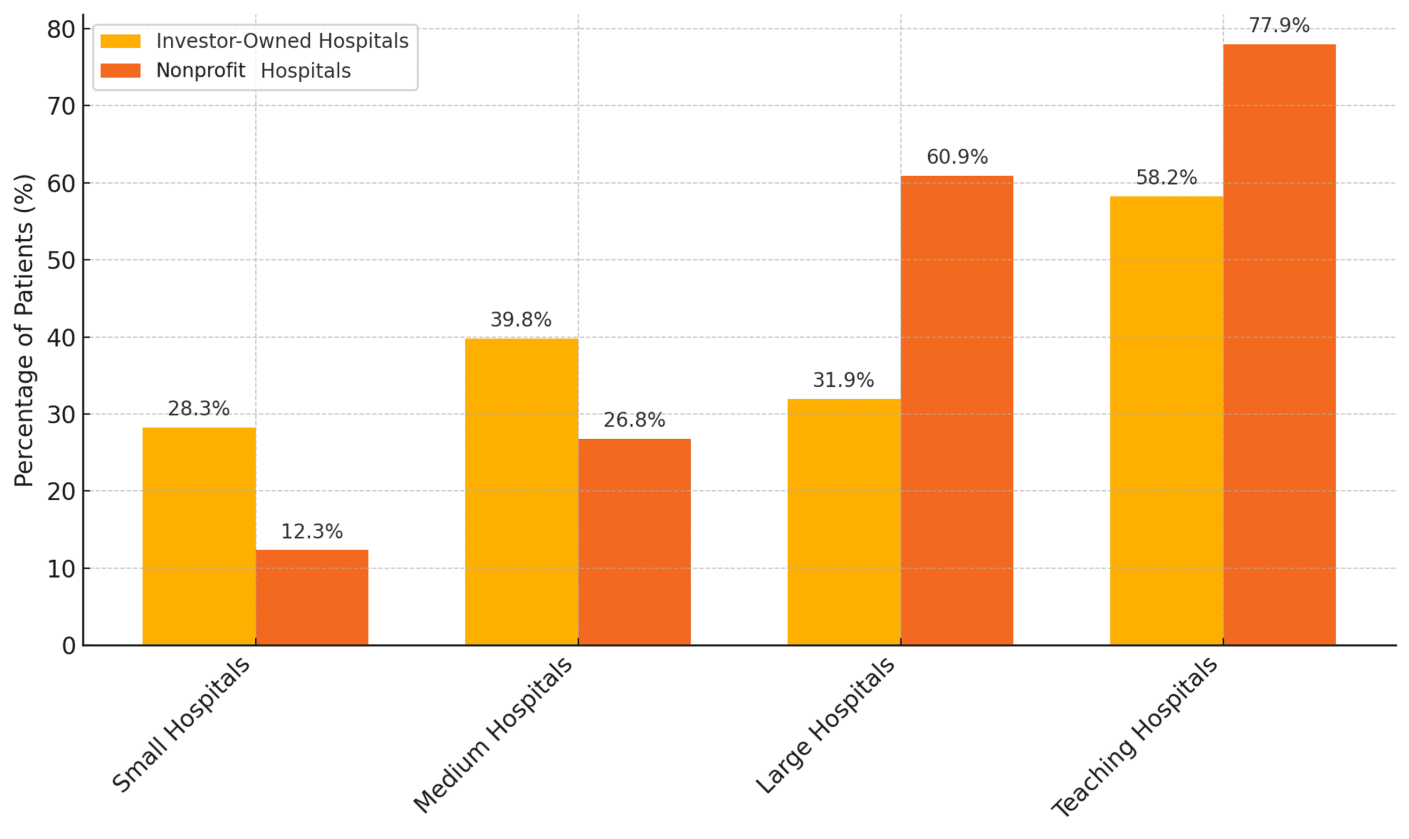
Distribution of hospital size and teaching status by ownership type.

The teaching status also differed: 77.94% of nonprofit hospitals were teaching institutions versus 58.24% of investor-owned (p < 0.001). Regional variation was significant (p < 0.001), with investor-owned hospitals more common in the South, while nonprofit hospitals were more represented in the Northeast and Midwest. Regional and institutional differences likely act as confounders and were adjusted for in all multivariate models (Figure 6).

Predictors of mortality

**Figure 7:**
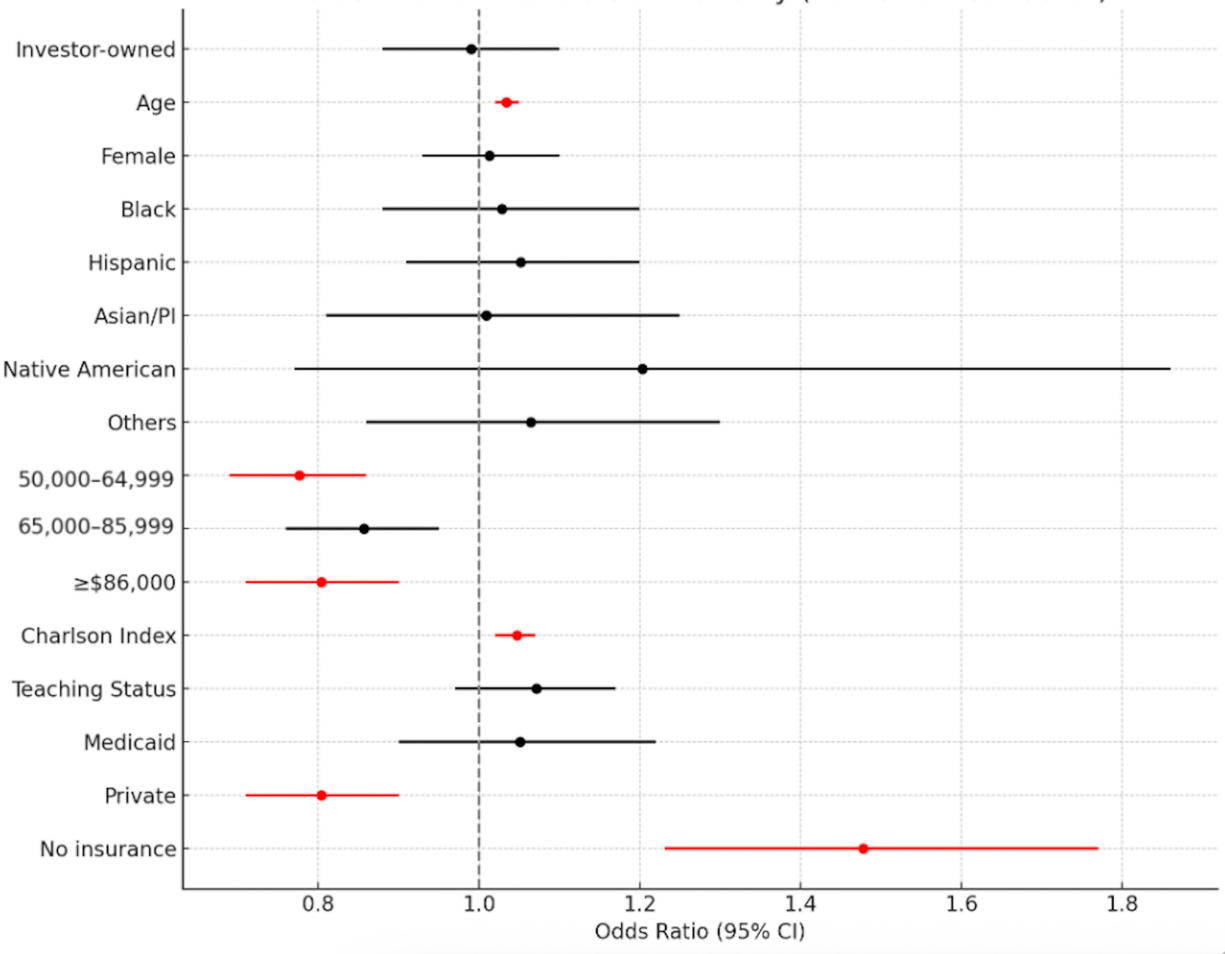
Forest plot of predictors of mortality with Bonferroni correction.

Table 4 and Figure 7 show multivariate mortality predictors. Hospital ownership was not a significant factor (odds ratio (OR) = 0.990, p = 0.863). Age was a strong predictor: each year increased odds of mortality by 3.4% (OR = 1.034, confidence interval (CI) = 1.02-1.03, p < 0.001), which is clinically meaningful in older populations. Female sex was not significant (OR = 1.013, p = 0.769). Higher income was protective, with significant reductions in mortality for all brackets above $50,000 (p < 0.006). The CCI was a consistent predictor, with a 4.7% increase in mortality odds per point (p < 0.001). Insurance status mattered: private insurance was protective (OR = 0.804), while no insurance increased risk (OR = 1.478, both p < 0.001). Teaching status was not a significant predictor (p = 0.146). Multicollinearity diagnostics were conducted and ruled out significant correlation among predictors, including income and insurance.

**Table 4:** Predictors of mortality. Comparisons were made using t-tests for continuous variables, with t-values ranging from -4.40 to 12.26. Statistical significance was defined as p-values <0.05. aOR = adjusted odds ratio; 95% CI = 95% confidence interval; PI = Pacific Islander; Ref = reference group; NI = not included; $ = Income range in U.S. dollars

Parameters	aOR	95% CI	P-value
Investor-owned	0.990	0.88-1.10	0.863
Age	1.034	1.02-1.03	<0.001
Female	1.013	0.93-1.10	0.769
Race/Ethnicity			
White	Ref	Ref	Ref
Black	1.028	0.88-1.10	0.725
Hispanic	1.052	1.02-1.03	0.473
Asian/PI	1.009	0.93-1.10	0.934
Native American	1.203	0.88-1.10	0.409
Others	1.064	1.02-1.03	0.552
Household income			
$1–$49,999	Ref	Ref	Ref
$50,000–$64,999	0.776	0.88-1.10	<0.001
$65,000–$85,999	0.857	1.02-1.03	0.006
≥$86,000	0.804	0.93-1.10	<0.001
Charlson Index	1.047	0.88-1.10	<0.001
Teaching status	1.071	1.02-1.03	0.146
Insurance			
Medicare	Ref	Ref	Ref
Medicaid	1.051	0.88-1.10	0.530
Private	0.804	1.02-1.03	<0.001
No insurance	1.478	0.93-1.10	<0.001

Predictors of length of stay

**Figure 8:**
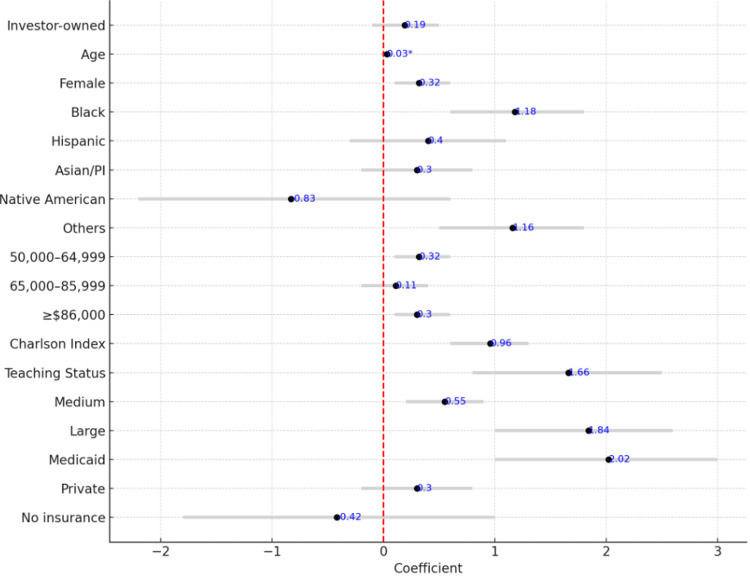
Forest plot of predictors of stay with Bonferroni correction.

Table 5 and Figure 8 present LOS predictors. Investor-owned status was not associated with LOS (coefficient = 0.19, p = 0.334). Older patients stayed slightly fewer days (coefficient = -0.03, p < 0.001), and females had shorter stays (coefficient = -0.32, p = 0.043). Black and “Other” race patients had significantly longer LOS than whites. While no insurance had a negative coefficient (-0.42), this was not statistically significant, though it may suggest a trend toward early discharge. LOS was longer in teaching hospitals (+1.66 days, p < 0.001) and large hospitals (+1.84 days, p < 0.001), which may reflect more complex case management and institutional practices.

**Table 5:** Predictors of length of stay. Comparisons were made using t-tests for continuous variables, with t-values ranging from -3.49 to 20.27. Statistical significance was defined as p-values <0.05. Coefficient = the estimated effect size of the variable on the outcome; 95% CI = confidence interval; Ref = reference group (used as the baseline comparison group in statistical analysis); LOS: length of stay; PI = Pacific Islander; NI: not included (indicates that the variable was not included in the statistical analysis); $ = income range in U.S. dollars

Parameters	Coefficient	95% CI	P-value
Investor-owned	0.19	-0.19 to 0.58	0.334
Age	-0.03	-0.05 to -0.02	<0.001
Female	-0.32	-0.63 to -0.01	0.043
Race/Ethnicity			
White	Ref	Ref	Ref
Black	1.18	0.54 to 1.82	<0.001
Hispanic	0.46	-0.18 to 1.11	0.163
Asian/PI	-0.30	-0.95 to 0.34	0.351
Native American	-0.83	-2.16 to 0.49	0.216
Others	1.16	0.15 to 2.16	0.024
Household income			
$1–$49,999	Ref	Ref	Ref
$50,000–$64,999	0.32	-0.06 to 0.72	0.103
$65,000–$85,999	0.11	-0.29 to 0.52	0.574
≥$86,000	0.36	-0.08 to 0.81	0.107
Charlson Index	0.96	0.87 to 1.05	<0.001
Teaching status	1.66	1.34 to 1.98	<0.001
Hospital bed size			
Small	Ref	Ref	Ref
Medium	0.51	0.09 to 0.93	0.017
Large	1.84	1.41 to 2.26	<0.001
Insurance			
Medicare	Ref	Ref	Ref
Medicaid	2.02	1.29 to 2.75	<0.001
Private	0.30	-0.10 to 0.71	0.142
No insurance	-0.42	-1.04 to 0.19	0.181

Predictors of hospital charges

**Figure 9:**
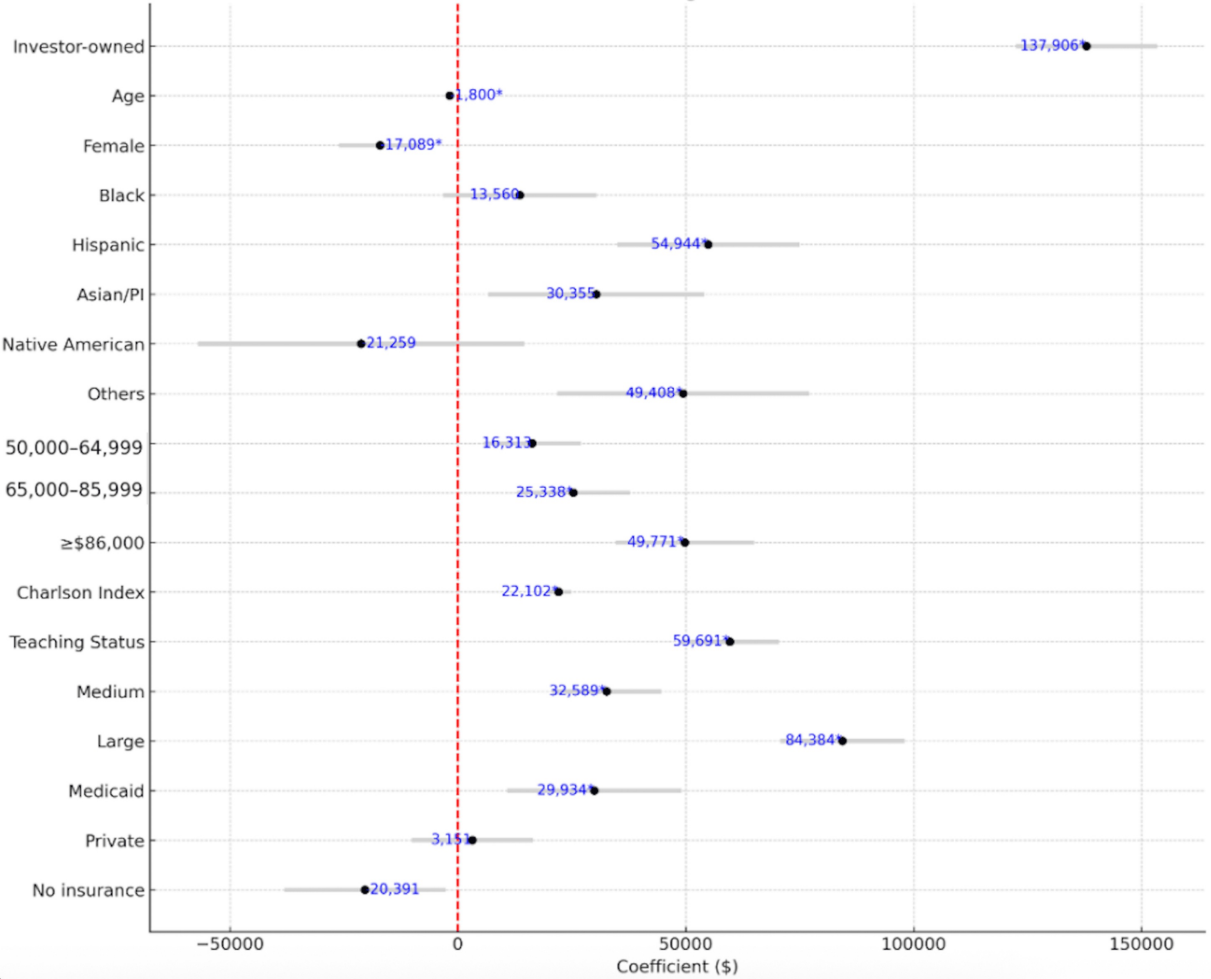
Forest plot of predictors of total charges with Bonferroni correction.

Hospital charges (Table 6, Figure 9) were significantly higher in investor-owned hospitals (+$137,906; p < 0.001), independent of LOS. Older age and female sex were associated with lower charges. Hispanic, Asian/Pacific Islander, and “Other” racial groups incurred significantly higher charges than white patients, possibly reflecting severity, delays in access, or disparities. Income level showed a stepwise increase in charges, possibly reflecting treatment aggressiveness, facility selection, or unmeasured confounding. Teaching hospitals (+$59,691) and larger hospitals were associated with higher charges. Medicaid was associated with significantly higher charges compared to Medicare (+$29,934), while uninsured patients had lower charges (-$20,391), possibly due to reduced billing or limited interventions. Notably, several predictors (e.g., female sex, CCI) influenced both LOS and charges, while others (e.g., hospital ownership) affected only charges, suggesting differences in billing strategies versus clinical utilization.

**Table 6:** Predictors of total charges. Comparisons were made using t-tests for continuous variables, with t-values ranging from -5.46 to 16.62. Statistical significance was defined as p-values <0.05. Coefficient: the estimated effect size of the variable on the outcome (likely a monetary value or length of stay in this context); 95% CI = confidence interval; Ref = reference group (used as the baseline comparison group in statistical analysis); PI = Pacific Islander; NI = not included (indicates that the variable was not included in the statistical analysis); $ = Income range in U.S. dollars

Parameters	Coefficient	95% CI	P-value
Investor-owned	137,906	122,366 to 153,446	<0.001
Age	-1,800	-2,286 to -1,314	<0.001
Female	-17,089	-26,156 to -8,021	<0.001
Race/Ethnicity			
White	Ref	Ref	Ref
Black	13,560	-3,220 to 30,340	0.113
Hispanic	54,944	34,944 to 74,944	<0.001
Asian/PI	30,355	6,661 to 54,049	0.012
Native American	-21,259	-57,050 to 14,531	0.244
Others	49,408	21,741 to 77,075	<0.001
Household income			
$1–$49,999	Ref	Ref	Ref
$50,000–$64,999	16,313	5,670 to 26,956	0.003
$65,000–$85,999	25,338	12,962 to 37,713	<0.001
≥$86,000	49,771	34,560 to 64,983	<0.001
Charlson Index	22,102	19,371 to 24,833	<0.001
Teaching status	59,691	48,974 to 70,408	<0.001
Hospital bed size			
Small	Ref	Ref	Ref
Medium	32,589	20,589 to 44,590	<0.001
Large	84,384	70,744 to 98,023	<0.001
Insurance			
Medicare	Ref	Ref	Ref
Medicaid	29,934	10,809 to 49,058	0.002
Private	3,151	-10,155 to 16,458	0.643
No insurance	-20,391	-38,066 to -2,716	0.024

## Discussion

CS as a result of STEMI is associated with significant morbidity and mortality. Our study aimed to identify whether hospital ownership influences outcomes in STEMI-associated CS. Mortality, our primary outcome, did not differ significantly between investor-owned and nonprofit hospitals, with an adjusted odds ratio (aOR) of 1.03 (p = 0.60; 95% CI = 0.91-1.16). This result remained consistent after PSM. As per the American Heart Association guidelines, CS management follows a stepwise approach beginning with vasopressors, such as dopamine, followed by mechanical circulatory support when indicated [17,18]. Early revascularization remains a cornerstone of STEMI treatment and may reduce the need for mechanical support [18]. Regardless of hospital ownership, adherence to guideline-directed care may account for the lack of observed mortality difference.

However, our study did identify patient-level predictors of mortality. Increasing age and uninsured status were associated with higher mortality. This is consistent with prior literature, where age-related physiologic decline, frailty, and comorbid burden contribute to poorer outcomes. Additionally, extracorporeal membrane oxygenation survival is notably lower among patients aged over 65 years [19,20]. Uninsured individuals may face barriers to timely care, receive fewer invasive interventions, and experience care limitations such as higher rates of do-not-resuscitate status or palliative referral, all contributing to worse outcomes [21]. These findings highlight the need for more equitable access to care and warrant further investigation.

Although mortality was not impacted by hospital ownership, significant variation in healthcare charges was observed. Investor-owned hospitals incurred substantially higher mean charges compared to nonprofit institutions (mean difference = $94,406; p < 0.01). Prior studies have shown that STEMI care costs vary with comorbidity severity, procedural complications, and intensity of care [22]. However, our dataset reflects billed charges, not true costs or resource use. These discrepancies may arise from differences in administrative pricing structures, billing practices, and regional cost factors rather than actual care delivery differences. This limitation of charge-based analysis must be acknowledged. Therefore, the observed cost differences between investor-owned and nonprofit hospitals may reflect differences in pricing and billing strategies rather than true variations in resource consumption. We acknowledge this distinction as a limitation of charge-based analyses.

LOS did not differ significantly between ownership groups. However, older patients had shorter LOS, which aligns with previous findings suggesting more rapid discharge or transfer to skilled care in the elderly [23]. The CCI was a strong predictor of mortality but was not significantly associated with LOS or total charges in our model. This contrasts with prior studies where CCI influenced both outcomes. The acute and protocolized nature of CS care, particularly in high-intensity settings, may mitigate the relative impact of comorbidities on LOS and charges. This contradiction merits further exploration.

Patient demographic analysis revealed that STEMI-CS admissions were predominantly white males on Medicare, consistent with prior literature [24-26]. Women and African Americans, despite lower representation, face disproportionately worse outcomes in CS [27]. These disparities may stem from underrecognition, treatment delays, and differential care practices, all of which deserve attention in future research. The statement that managing minority groups remains a challenge is supported by evidence demonstrating care inequities and implicit bias.

Most patients in our cohort were treated in large, urban teaching hospitals. Teaching institutions often have multidisciplinary teams and greater infrastructure to manage CS. Previous studies show higher adherence to guidelines and better process-of-care metrics in teaching hospitals [28]. However, we found no mortality advantage in these settings. This may suggest that in high-acuity conditions such as CS, differences in care processes may not translate to survival benefits, potentially due to the overwhelming severity of illness at presentation.

Hospital-level characteristics such as size, teaching status, and regional location varied by ownership and may act as confounders. Investor-owned hospitals had more patients treated in smaller facilities, which may be located in rural or suburban areas. These structural and geographic factors could influence access, staffing, and timeliness of interventions such as catheterization. We adjusted for these variables in our models and clarified that hospital characteristics were analyzed at the patient level. This may reflect structural and geographic factors, including the distribution of investor-owned hospitals in regions with higher poverty rates or limited access to nonprofit institutions [29,30].

Several limitations should be acknowledged. First, our study used ICD-10-CM coding algorithms to identify STEMI-induced CS and PCI procedures. This approach, while consistent with prior studies using administrative datasets, may introduce misclassification bias, which we acknowledge as a limitation. The full list of ICD-10-CM and ICD-10-PCS codes used is provided in the Appendices. Second, the NIS database does not provide direct measures of clinical severity, such as hemodynamic parameters or shock stage, nor does it capture procedural timing. These unmeasured variables could impact mortality and resource use. Third, we were unable to assess treatment adherence or procedural quality, and our assumption that care was guideline-concordant across hospital types remains speculative. Fourth, although listwise deletion was used to handle missing data, we acknowledge that this approach assumes data are MCAR, and no sensitivity analyses were conducted. The total number and percentage of excluded records are reported in the Results section. Fifth, transfers between hospitals could not be reliably excluded, which may impact mortality and cost estimates. Finally, we reiterate that NIS captures billed charges rather than actual costs; differences in charges may reflect pricing and administrative practices rather than resource utilization.

## Conclusions

The rising trend of private-equity hospitals in the US, often hypothesized to emphasize operational efficiency and revenue generation, has led to higher patient charges compared with nonprofit institutions. In our retrospective analysis of six years of NIS data on CS hospitalizations, we observed no significant differences in mortality or LOS despite these increased charges at investor-owned hospitals. As an observational, hypothesis-generating study, these results highlight a critical gap in the literature on treatment outcomes for CS by hospital ownership and suggest that physicians and policymakers should reassess whether elevated charges translate into enhanced patient care or primarily reflect profit-driven billing practices.

## Figures and Tables

**Figure 10:**
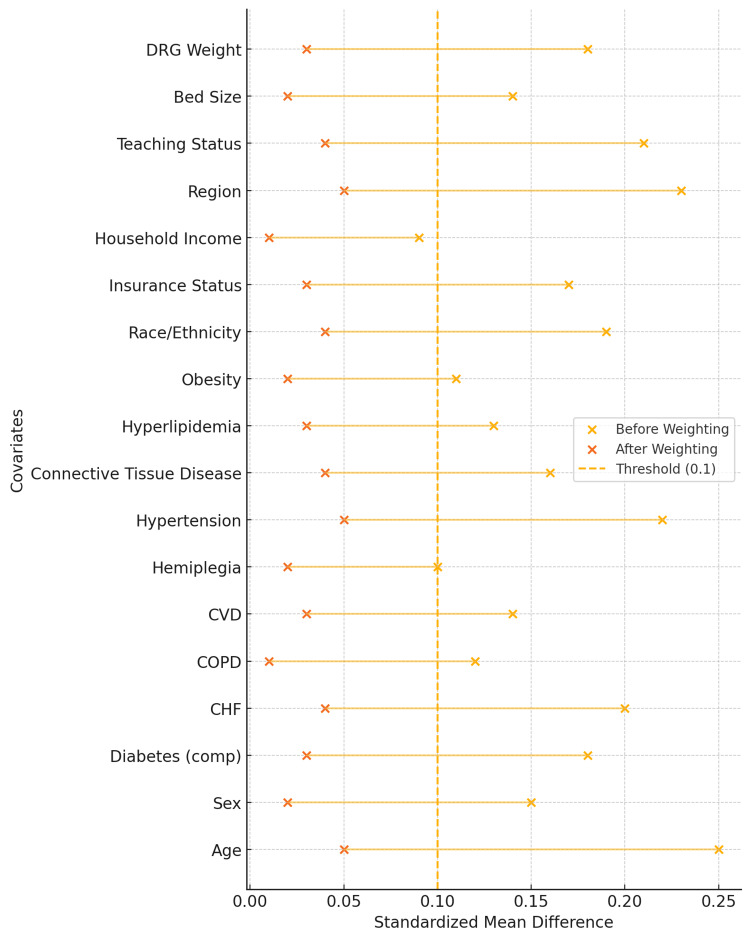
Covariate balance before and after weighting. Love plot showing standardized mean differences (SMDs) for each covariate before (orange circles) and after (red crosses) inverse-probability weighting. Horizontal lines connect pre- and post-weighting SMDs for each variable. The dashed vertical line at 0.1 represents the threshold below which covariate balance is generally considered acceptable. DRG = diagnosis-related group; CVD = cerebrovascular disease; COPD = chronic obstructive pulmonary disease; CHF = congestive heart failure

## Appendices

**Table 7:** ICD-10 codes used to define study population. ICD-10 = International Classification of Diseases, Tenth Revision; STEMI = ST-elevation myocardial infarction; DES = drug-eluting stent

Category	ICD-10 code	Description
Diagnosis	R57.0	Cardiogenic shock
Diagnosis	I21.0	STEMI, left main coronary artery
Diagnosis	I21.1	STEMI, left anterior descending artery
Diagnosis	I21.2	STEMI, other sites
Diagnosis	I21.3	STEMI, unspecified site
Procedure	02703ZZ	Dilation of coronary artery, one site, DES
Procedure	02713ZZ	Dilation of coronary artery, two sites, DES
Procedure	02723ZZ	Dilation of coronary artery, three sites, DES
Procedure	02733ZZ	Dilation of coronary artery, four or more sites, DES
